# A Double-Blind Placebo-Controlled Randomized Trial Evaluating the Effect of Polyphenol-Rich Herbal Congee on Bone Turnover Markers of the Perimenopausal and Menopausal Women

**DOI:** 10.1155/2018/2091872

**Published:** 2018-11-21

**Authors:** Jintanaporn Wattanathorn, Woraluk Somboonporn, Sudarat Sungkamanee, Wipawee Thukummee, Supaporn Muchimapura

**Affiliations:** ^1^Department of Physiology, Faculty of Medicine, Khon Kaen University, Khon Kaen 40002, Thailand; ^2^Integrative Complementary Alternative Medicine Research and Development Center, Khon Kaen University, Khon Kaen 40002, Thailand; ^3^Department of Obstetrics and Gynaecology, Faculty of Medicine, Khon Kaen University, Khon Kaen 40002, Thailand; ^4^Department of Physiology (Neuroscience Program), Faculty of Medicine, Khon Kaen University, Khon Kaen 40002, Thailand

## Abstract

Based on the benefit of polyphenolic compounds on osteoporosis, we hypothesized that the polyphenol-rich herbal congee containing the combined extract of *Morus alba* and *Polygonum odoratum* leaves should improve bone turnover markers in menopausal women. To test this hypothesis, a randomized double-blind placebo-controlled study was performed. A total of 45 menopausal participants were recruited in this study. They were randomly divided into placebo, D1, and D2 groups, respectively. The subjects in D1 and D2 groups must consume the congee containing the combined extract of *M. alba* and *P. odoratum* leaves at doses of 50 and 1500 mg/day, respectively. At the end of an 8-week consumption period, all subjects were determined serum bone markers including calcium, alkaline phosphatase, osteocalcin, and beta CTX. In addition, the hematological and blood clinical chemistry changes, and total phenolic content in the serum were also determined. The results showed that the menopausal women in D2 group increased serum alkaline phosphatase, osteocalcin, and total phenolic compounds content but decreased CTX level. Clinical safety assessment failed to show toxicity and adverse effects. Therefore, herbal congee containing the combined extract of *M. alba* and *P. odoratum* leaves is the potential functional food that can decrease the risk of osteoporosis.

## 1. Introduction

Bone is a dynamic organ that undergoes continuous remodeling by the coordination and balance between resorption and the formation activities of osteoclast and osteoblast cells [1]. It is well established that women are vulnerable to bone loss especially during and after menopause. Postmenopausal women lose trabecular bone mineral density (BMD) rapidly in their vertebrae, pelvis, and ultradistal wrist. After menopause, the cortical bone loss in the long bones and vertebrae also occurs, but the rate of bone loss is slower than that in trabecular bone [2]. It has been reported that bone resorption assessed by using the bone resorption markers as indicator increases around 90% during this period [3]. Therefore, this situation increases bone fracture risk.

Currently, a noninvasive instrument such as dual energy X-ray absorptiometry (DXA) can be used as a tool to measure the body composition including bone density. However, it still has a limitation, and bone markers are more sensitive for detecting the change of bone dynamic [4]. Therefore, the application of bone turnover markers (BTM) as the indices indicating bone dynamic has gained much attention. BTM are classified as the formation or resorption markers depending on their origins. It has been demonstrated that BTM changes occur in parallel with bone turnover process, so their alterations can reflect bone turnover state. Among various BTM, osteocalcin and alkaline phosphatase are regarded as bone formation markers, whereas C-telopeptide of type I collagen (CTX) and N-telopeptide of type I collagen (NTX) and tartrate-resistant acid phosphatase (TR-ACP) are regarded as bone resorption markers [5–8]. Recent studies have clearly demonstrated that polyphenolic compounds provide beneficial effect to bone health by decreasing oxidative stress and inflammation. In addition, they can also modulate osteoblastogenesis and osteoclastogenesis [9]. Based on the previous finding in experimental menopause that the combined extract of *Morus alba* and *Polygonum odoratum* showed the antiosteoporotic effect [10], the positive modulation effect of the congee containing the combined extract of *P. odoratum* and *M. alba* on bone turnover has been raised.

However, recent study has demonstrated that polyphenol substance can exert prooxidant activity [11] and interactions with other agents which in turn produces numerous detrimental effects [12]. Therefore, the adverse effects and toxicities can possibly occur especially in the repetitive administration of the polyphenol-rich product. To assure that the repetitive administration of the congee containing the combined extract of *P. odoratum* and *M. alba* exert the positive modulation effect on the bone turnover and safe for repetitive consumption, the effect of the congee mentioned earlier on bone formation and bone resorption markers together with the hematological and clinical chemistry values in the perimenopausal and menopausal women were determined.

## 2. Materials and Methods

This study was designed as the randomized controlled trial. All experiments were conducted at the Integrative Complementary Alternative Medicine Research and Development Center, Faculty of Medicine, Khon Kaen University. The study (code number UAPSBS201401) was conducted in accordance with the International Conference of Harmonization (ICH) for Good Clinical Practice (GCP) and in compliance with the Declaration of Helsinki and its further amendments. All protocols were approved by the Khon Kaen University Ethical Committee on Human Research (HE571373); ClinicalTrials.gov was approved ID NCT02562274.

### 2.1. Participants

Forty-five healthy Thai perimenopausal and postmenopausal women at the age between 45–60 years old (<5 years menstruation cessation) who lived in northeastern of Thailand were recruited to participate this study via advertisements at menopause clinic at Srinagarind Hospital, Faculty of Medicine, Khon Kaen University, Thailand. All subjects in this study were subjected to a physical examination and interviewed using a structured questionnaire to obtain information concerning demographic data, smoking, alcohol intake, physical activity, and dietary status. Subjects were excluded from this study if they were diagnosed with hypertension, cancer, autoimmune diseases, gout or high uric acid, and disorders as described as follows: heart, liver, kidney, lung, mental, and endocrinological disorders such as thyroid, parathyroid, and diabetes. In addition, they should not have a history of drug use that could affect bone turnover and bone mineral density, pesticide exposure during one week before the test, hysterectomy and/or oophorectomy, alcohol or cigarettes addiction (cigarettes smoking >10 pieces/day), regular exercise (more than 3 time/weeks), unable to follow the study instructions during the trial and the participation in other projects.

### 2.2. A Polyphenol-Rich Congee Preparation

**Figure 1:**
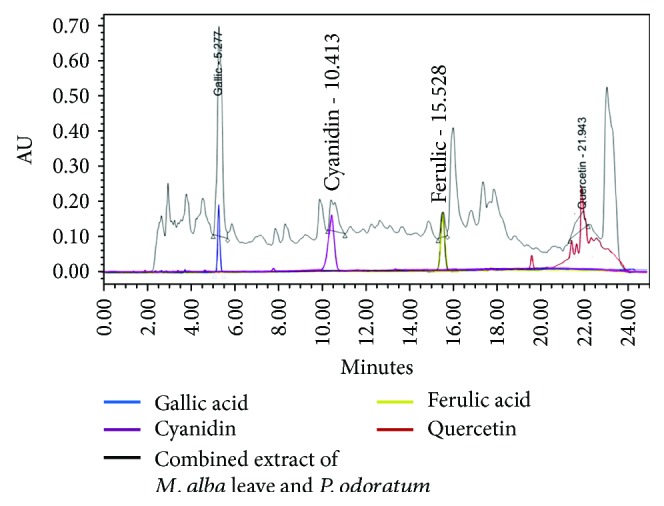
Fingerprint chromatogram of herbal congee containing the combined extract of *M. alba* and *P. odoratum.*

The aerial parts of *Polygonum odoratum* and *M. alba* leaves were collected from Amphoe Muaeng Khon Kaen, Thailand, during April 2013 and prepared as water extract by decoction method. Then, they were filtered and lyophilized as powder. The combination extract of *P. odoratum* and *M. alba* was prepared according to petty patent number 9314, Department of Intellectual Property, Thailand, and used as the functional ingredient in herbal porridge. Each package (25 g/package) contained rice (dried mashed) 90.71%, chicken (dried mashed) 7.26%, and green shallot and coriander (dried mashed) 0.73%. Both placebo and herbal congee were prepared with the same procedure and contained the same ingredients except that the herbal congee contained the combined extract of *P. odoratum* and *M. alba* at doses of 50 and 1500 mg/pack, respectively (the selected doses were obtained from our preclinical data [10] by calculating the human equivalent doses). The placebo, D1, and D2 had the same appearance and smell. The congee containing high and low doses of the combined extract of *P. odoratum* and *M. alba* failed to show the significant calorie from placebo (placebo provided 94.24 whereas the herbal congee containing low and high doses of the combined extract of *P. odoratum* and *M. alba* provided 95.50 and 97.25 Kcal, respectively). Each package contained carbohydrate 20 grams, protein 2 grams, total fat 0 gram, cholesterol 0 mg, and fiber less than 1 gram. In addition, the fingerprint chromatogram of the herbal congee was determined via high performance liquid chromatography which consisted of 515 HPLC pump and 2998 photodiode array detector (Water Company, USA). Chromatographic separation was performed using C-18 endcapped Purospher® STAR column (250 × 4 mm; particle size; 5 *μ*m) and guard column HPLC-Cartridge, Sorbet Lot No. HX255346 (Merck, Germany). Two mobile phases consisting of 2.5% acetic acid in deionized (DI) water (B) and methanol (A) were used to induce gradient elution. The gradient elution was carried out at a flow rate of 1.0 mL/min with the following gradient: 0 min, 10% A; 17 min, 70% A; 18–22 min, 100% A; 25, 50% A; 26–30 min, 10% A. The sample was filtered (0.2 *μ*m, Whatman), and a direct injection of tested sample at the volume of 20 *μ*L on the column was performed. The chromatograms were recorded at 280 nm using UV detector, data analysis was performed using EmpowerTM3, and the results were shown in Figure 1.

### 2.3. Experimental Design

Eligibility was evaluated by using the semistructure questionnaire, physical examination, and the results of the prestudy laboratory tests. Then, they were randomly allocated into placebo group and D1- and D2-treated groups. Subjects in D1- and D2-treated groups must consume the herbal congee containing the combined extract of *P. odoratum* and *M. alba* at doses of 50 and 1500 mg/day, respectively. Prior to the study participation, the participants must fill the informed consent form.

On the experimental day, all participants should not drink tea or coffee or alcohol beverages at least 12 hours before their appointments. In addition, the participants must consume the assigned substance once daily in the morning before breakfast for 8 weeks in a double-blind fashion. All subjects collected blood for the determination of bone markers consisting of serum levels of calcium, osteocalcin, alkaline phosphatase, and beta CTX together with the safety parameters including hematological and clinical chemistry changes prior to the study and at the end of an 8-week study period.

All volunteers were kept blind about treatments. The data interpretation and analysis were performed by blinded investigators, and the code numbers and the group allocation were revealed after the assessment of the last subject. All volunteers were instructed to call the study center in case of any adverse effect occurred during the study. Volunteers had the opportunity to withdraw from the study at any time. All the volunteers were contacted at definite intervals to ensure that they consumed the assigned substance regularly.

### 2.4. Blood Collection

Blood samples were collected from the participants after an overnight fast by venipuncture and allowed to clot and spun at 3000 rpm for 10 minutes in order to separate cells from serum. Then, the serum was transferred into dry well-labeled specimen plastic tubes and analyzed.

### 2.5. Determination of Bone Markers

The assessment of serum osteocalcin concentration was carried out by using human Gla-OC High sensitive EIA Kit with the intra- and interassay coefficient of variations (CVs) <5%. In brief, an aliquot of 100 *μ*L standard or sample was added to an appropriate well and incubated at room temperature for 2 hours. Then, the sample solution was removed, and the well was washed 3 times with 400 *μ*L of PBS. 100 *μ*L total of antibody-POD conjugate solution was added into the wells and incubated for 1 hour. At the end of the incubation period, the solution was aspirated from the wells, and the wells were washed again for 4 times with washing buffer (100 *μ*L/well). After this step, the substrate solution at the volume of 100 *μ*L was added to each well and incubated at room temperature for 15 minutes. At the end of incubation period, 100 *μ*L of the stop solution was added into all wells and mixed gently. Then, an absorbance at 450 nm was recorded via microplate reader. All assays were performed in triplicate.

In this study, the concentration of beta-carboxy-terminal cross-linking telopeptide of type I collagen (CTX) levels was measured by using commercial human Micro ELISA Kit with intra- and interassay coefficients of variations 8 and 10%, respectively. In brief, all reagents and samples were left at room temperature before used. After thawing, samples were centrifuged again before the assay. All the reagents were mixed thoroughly by gently swirling before pipetting. An aliquot of 50 L of biotinylated detection Ab working solution was added to each well, covered with plate sealer, gently tapped to mix a solution, and incubated at 37°C for 45 minutes. Each well was aspirated and washed with wash buffer for three times. After washing, the remaining wash buffer was removed by aspirating, and the plate was inverted and patted against thick clean absorbent paper. Then, 100 *μ*L of HRP conjugate working solution was added to each well, covered with a new plate sealer, and incubated at 37°C for 30 minutes. After the incubation, washing was performed for five times. Then, 90 *μ*L of substrate solution was added to each well, covered with a new plate sealer, and incubated at 37°C for 15 minutes with light protection. The reaction time can be shortened or extended according to the actual color change, but not more than 30 minutes. Then, add 50 *μ*L of stop solution to each well and the optical density at 450 nm was monitored via microplate reader.

### 2.6. Determination of Total Phenolic Compounds

The total phenolics compounds of vegetables were measured by using Folin-Ciocalteu colorimetric method (Quettier-Deleu et al., 2000). Briefly, 20 *μ*L of each plant extracts was mixed with 0.2 mL of Folin-Ciocalteu reagent and 2 mL of distilled water and incubated at room temperature for 5 minutes. Then, 1 mL of 20% sodium carbonate was added and incubated at room temperature for 2 hours. The total polyphenolic compounds were determined by measuring the absorbance at 765 nm with spectrophotometer. Gallic acid was used as a standard, and the total phenolics were expressed as gallic acid equivalents (mg/L GAE/mg extract). All determinations were performed in triplicate.

### 2.7. Determination of Calcium and Alkaline Phosphatase in Serum

Blood was collected, and the determinations of calcium and alkaline phosphatase changes were performed at Srinagarind Hospital, Faculty of Medicine, Khon Kaen University, Khon Kaen, Thailand. The coefficient of variation (% CV) of calcium (Ca2+) and alkaline phosphatase (ALP) was 1.7% and 0.6%, respectively.

### 2.8. Blood Collection and Toxicity Assessment

Blood was taken from each volunteer and prepared as plasma in order to assess the changes of hematological and clinical chemistry parameters. All parameters were assessed at Srinagarind Hospital.

### 2.9. Statistical Analysis

All data were expressed as mean ± SD. Comparisons between groups were performed using one-way analysis of variance (ANOVA) followed by post hoc (LSD) multiple comparison tests and Kruskal-Wallis one-way analysis of variance test by using SPSS statistical software. *P* value < 0.05 was considered significant.

## 3. Results

### 3.1. Demographic Data of Subjects

**Figure 2:**
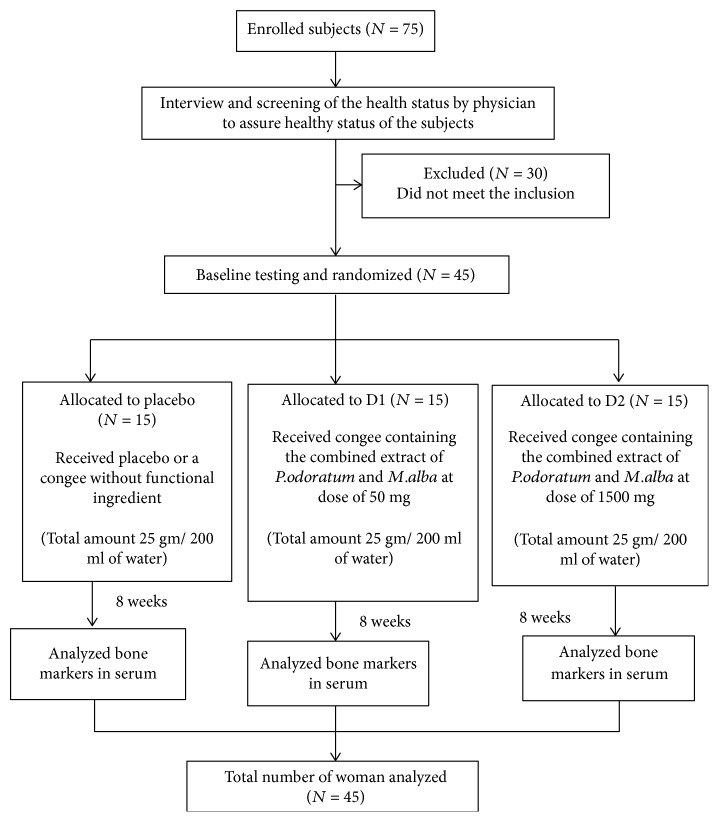
Flow diagram of subjects.

Seventy-five menopause women were enrolled to participate in this study via the advertisements at menopause clinic, Srinagarind Hospital, Faculty of Medicine, Khon Kaen University, Khon Kaen, Thailand. After the interview and the screening of health status by the physician, it was found that only 45 subjects met the inclusion criteria and were allocated to placebo, D1, and D2 groups as shown in Figure 2. The baseline demographic data of all participants were presented in Table 1. No significant differences in all parameters were observed.

### 3.2. Effect of Herbal Congee on Bone Makers

**Figure 3:**
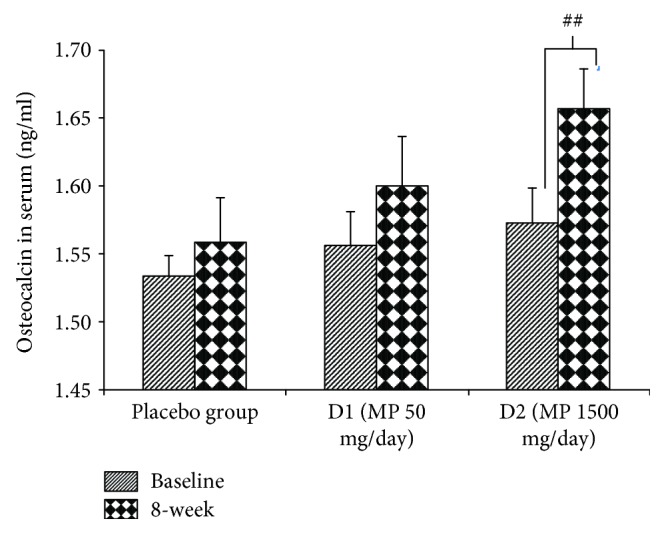
The effect of various doses of the herbal congee containing the combined extract of *M. alba* leaves and *P. odoratum* on the serum osteocalcin level (*n* = 15/group) ^##^*P* value < 0.01; compared to baseline. D1 = MP 50 mg/day; D2 = MP 1500 mg/day.

**Figure 4:**
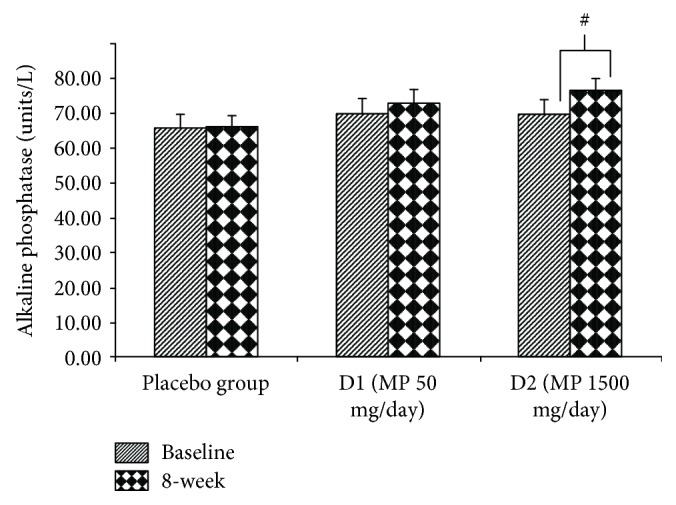
The effect of various doses of the herbal congee containing the combined extract of *M. alba* leaves and *P. odoratum* on the level of alkaline phosphatase in serum (*n* = 15/group) ^#^*P* value < 0.05; compared to baseline. D1 = MP 50 mg/day; D2 = MP 1500 mg/day.

**Figure 5:**
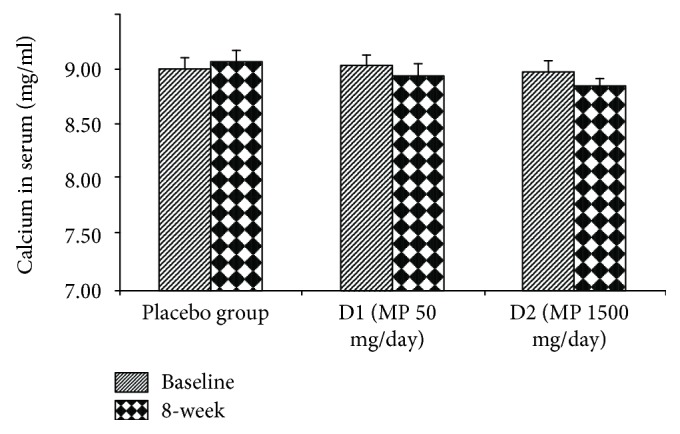
The effect of various doses of the herbal congee containing the combined extract of *M. alba* leaves and *P. odoratum* on the level of calcium in serum (*n* = 15/group). D1 = MP 50 mg/day; D2 = MP 1500 mg/day.

**Figure 6:**
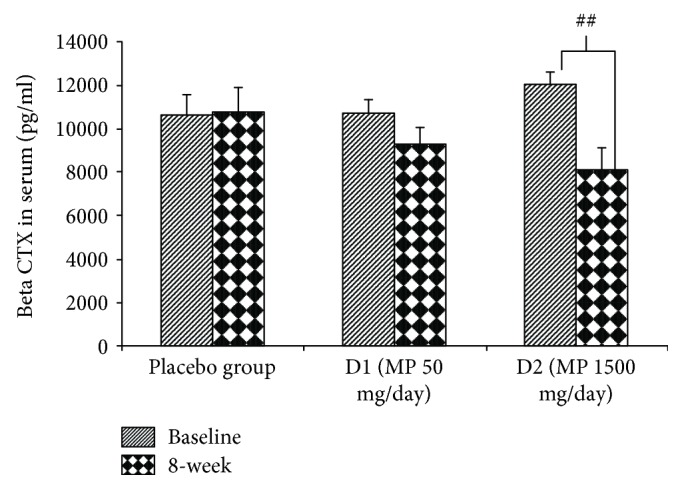
The effect of various doses of the herbal congee containing the combined extract of *M. alba* leaves and *P. odoratum* on the level of serum beta CTX (*n* = 15/group). ^##^*P* value < 0.01; compared to baseline. D1 = MP 50 mg/day; D2 = MP 1500 mg/day.

The effect of various doses of the herbal congee containing the combined extract of *M. alba* leaves and *P. odoratum* on the level of osteocalcin, alkaline phosphatase, calcium, and beta CTX in serum was shown in Figures 3–6. Subjects who consumed the herbal congee containing the combined extract of *M. alba* leaves and *P. odoratum* showed the elevations of serum osteocalcin and alkaline phosphatase levels (*P* value < 0.01and 0.05; compared to placebo group) as shown in Figures 3 and 4. However, the serum calcium level failed to show the significant change, while the serum beta CTX showed the significant reduction (*P* value < 0.01; compared to placebo group) as shown in Figures 5 and 6, respectively.

### 3.3. Effect of Herbal Congee on the Total Phenolic Compounds

**Figure 7:**
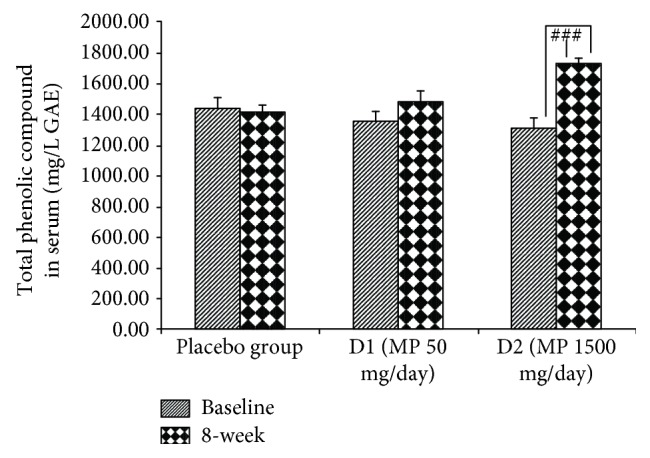
The effect of various doses of the herbal congee containing the combined extract of *M. alba* leaves and *P. odoratum* on the level of phenolic compounds in serum of all subjects (*n* = 15/group) ^###^*P* value < 0.001; compared to baseline. D1 = MP 50 mg/day; D2 = MP 1500 mg/day.

The effect of various doses of the herbal congee containing the combined extract of *M. alba* leaves and *P. odoratum* on the level of total phenolic compounds was shown in Figure 7. It was found that after 2 months of administration, subjects who consumed the herbal congee containing the combined extract of *M. alba* leaves and *P. odoratum* at dose of 1500 mg per day showed the significant elevation of total phenolic compound (*P* value < 0.001; compared to placebo, *P* value < 0.001; compared to baseline level).

### 3.4. Effect of Herbal Congee on the Blood Chemistry Parameters

Table 2 showed the effect of various doses of herbal congee containing the combined extract of *M. alba* leaves and *P. odoratum* on blood chemistry values. It was found that no significant differences of the parameters were observed at baseline level. At the end of an 8-week consumption period, subjects who consumed herbal congee containing the combined extract of *M. alba* leaves and *P. odoratum* at doses of 50 and 1500 mg per day showed the significant reduction of triglyceride (*P* value < 0.01and 0.05, respectively; compared to placebo group) together with the elevations of albumin (*P* value < 0.05; compared to placebo-treated group). In addition, the elevation of high-density lipoprotein cholesterol (HDL-C) was also observed in subjects who consumed the high dose of the herbal congee at the end of experimental period (*P* value < 0.05; compared to placebo-treated group).

### 3.5. Effect of Herbal Congee on the Hematological Changes

Table 3 showed the effect of various doses of herbal congee containing the combined extract of *M. alba* leaves and *P. odoratum* on the hematological values. It was found that at baseline consumption period, no significant changes of all parameters were observed. At the end of an 8-week consumption period, subjects who consumed herbal congee containing the combined extract of *M. alba* leaves and *P. odoratum* at dose of 1500 mg per day showed the significant reduction of platelets (*P* value < 0.05; compared to placebo group). However, the changes of all parameters were also in the normal range. No changes of other parameters were observed.

## 4. Discussion

This study clearly revealed that no major side effects or clinically significant symptoms were reported from any of the volunteers. No data of clinical chemistry and hematological values showed the significant toxicity. Therefore, these data support the consumption safety for healthy menopausal women. In addition, this study clearly revealed that subjects who consumed the herbal congee containing the combined extract of *M. alba* leaves and *P. odoratum* at dose of 1500 mg per day increased the total phenolic compounds in serum and improved bone formation markers including osteocalcin and alkaline phosphatase (ALP) but decreased bone resorption marker including serum collagen type 1 cross-linked C-telopeptide (beta CTx).

Osteoblasts which are responsible for bone formation are located on the bone surface. Bone formation is enhanced by the osteoproduction action of osteoblasts. During bone formation process, alkaline phosphatase is produced by osteoblasts during the synthesis of the collagen matrix [13]. In addition to alkaline phosphatase, osteocalcin, a 5.8 kDa hydroxyapatite-binding bone-specific protein, is also produced by osteoblasts [14]. In contrast to both parameters mentioned earlier, beta CTx is released into the bloodstream during bone resorption and serves as a specific marker for the degradation of mature type I collagen. Therefore, an elevated serum concentration of beta-CTx is reported to reflect bone resorption [15]. It has been reported that the rapid increase in serum beta CTX concentration is related to the secretion activity of osteoclasts [16]. The current data showed the elevation of alkaline phosphatase and osteocalcin but decreased beta-CTx level in serum of subjects who consumed MP at dose of 1500 mg/day. These results indicated the positive modulation effect of the herbal congee on the bone turn over which enhanced the bone formation but decreased bone resorption. Our data were in agreement with the previous study which showed the positive modulation effect of the combined extract of *M. alba* and *P. odoratum* (MP) on bone turnover in animal model of menopause [10].

Recent finding has demonstrated that polyphenolic compounds inhibit receptor activator of nuclear factor-*κ*B ligand- (RANKL-) induced osteoclast formation [17]. Since the herbal congee containing the combination extract of *P. odoratum* and *M. alba* contained high concentration of polyphenol, this substance may possibly be active constituent which contributes a role on an antiosteoclastogenic effect of the herbal congee containing the combination extract of *P. odoratum* and *M. alba*. However, further researches concerning depth analysis for active ingredient and the detail mechanism of action are still essential.

Although the current results clearly showed that the congee containing *P. odoratum* and *M. alba* is safe for consumption, the cholesterol showed the increasing trends. Therefore, the long-term application of the product should be a caution about hypercholesterolemia. However, studies which focused on the effect of long-term treatment should also be performed in order to assure that no side effect on hypercholesterolemia occurs. In addition, drug interaction is also possible during long-term application especially the interaction with the drugs commonly used in daily life such as paracetamol. Thus, the information concerning this aspect still requires further researches. The limitation of this study is that no data concerning the bone density can be demonstrated because the treatment duration is not long enough to produce the changes of bone density.

## 5. Conclusion

The present study demonstrates the antiosteoporotic effect of the polyphenol-rich herbal congee which contained the combined extract of *P. odoratum* and *M. alba.* The possible underlying mechanism may occur via the improved bone turnover via the increased bone formation and the decreased bone resorption. Therefore, the herbal congee containing the combined extract of *P. odoratum* and *M. alba* may be useful for the prevention and treatment of osteoporosis in menopause. However, long-term treatment study is still required to assure that no side effect on hypercholesterolemia.

## Figures and Tables

**Table 1:** The demographic data of subjects. Data were presented as mean *±* SD (*n* = 15/group).

Parameters	Placebo	MP 50 mg/day	MP1500 mg/day	*P* value
Age (years)	51.41 ± 4.21	50.47 ± 3.20	50.47 ± 3.64	0.697
Education (years)	7.73 ± 4.89	7.73 ± 4.13	7.47 ± 4.50	0.983
Body mass index (kg/m^2^)	24.27 ± 2.91	25.23 ± 3.52	24.91 ± 3.81	0.742
Blood sugar (mg/dL)	87.33 ± 13.50	84.67 ± 6.89	88.93 ± 15.04	0.635
Uric acid (mg/dL)	5.34 ± 0.77	5.05 ± 0.76	5.43 ± 0.92	0.422
Heart rate (beats/min)	77.00 ± 12.86	73.20 ± 9.20	74.27 ± 8.66	0.455
Respiratory rate (times/min)	19.80 ± 2.40	18.00 ± 1.98	18.80 ± 1.82	0.070
Systolic blood pressure (mmHg)	115.80 ± 11.48	118.67 ± 13.84	119.13 ± 13.06	0.745
Diastolic blood pressure (mmHg)	75.87 ± 8.93	78.00 ± 9.38	76.33 ± 8.47	0.791

**Table 2:** Effect of various doses of herbal congee containing the combined extract of *M. alba* leaves and *P. odoratum* or MP on blood clinical chemistry values (*N* = 45). Values are expressed as mean ± SD; ^∗^*P* value < 0.05, ^∗∗^*P* value < 0.01; compared to placebo group.

Blood chemistry	Normal value	Group	Baseline	8 weeks
			Mean ± SD	Mean ± SD
Glucose (mg/dL)	70–100 mg/dL	Placebo	87.33 ± 13.50	88.27 ± 12.19
		MP 50 mg/day	84.67 ± 6.89 F (1,28) = 0.00, *P* = 0.983	81.47 ± 7.79 F (1,28) = 2.777, *P* = 0.096
		MP 1500 mg/day	88.93 ± 15.04 F (1,28) = 0.23, *P* = 0.989	88.60 ± 15.80 F (1,28) = 2.814, *P* = 0.245

Uric acid (mg/dL)	2.7–7.0 mg/dL	Placebo	5.34 ± 0.77	4.96 ± 0.81
		MP 50 mg/day	5.05 ± 0.76 F (1,28) = 1.097, *P* = 0.304	4.53 ± 1.08 F (1,28) = 0.656, *P* = 0.418
		MP 1500 mg/day	5.43 ± 0.92 F (1,28) = 0.078, *P* = 0.782	4.74 ± 1.17 F (1,28) = 0.622, *P* = 0.733

Bicarbonate (mEq/L)	20.6–28.2 mEq/L	Placebo	26.41 ± 1.78	25.07 ± 1.34
		MP 50 mg/day	27.68 ± 2.56 F (1,28) = 0.520, *P* = 0.477	24.79 ± 1.99 F (1,28) = 0.124, *P* = 0.724
		MP 1500 mg/day	27.07 ± 2.42 F = (1,28) = 0.741 *P* = 0.37	24.11 ± 3.24 F = (1,28) = 0.149 *P* = 0.928

Blood urea nitrogen (mg/dL)	20.6–28.2 mEq/L	Placebo	11.40 ± 2.73	11.53 ± 2.57
		MP 50 mg/day	11.61 ± 3.18 F (1,28) = 0.039, *P* = 0.085	12.19 ± 2.57 F (1,28) = 0.496, *P* = 0.487
		MP 1500 mg/day	10.89 ± 2.39 F (1,28) = 0.292, *P* = 0.593	11.41 ± 2.87 F (1,28) = 0.013, *P* = 0.910

Creatinine (mg/dL)	0.5–1.5 mg/dL	Placebo	0.66 ± 0.09	0.69 ± 0.10
		MP 50 mg/day	0.68 ± 0.11 F (1,28) = 0.538, *P* = 0.463	0.68 ± 0.08 F (1,28) = 0.072, *P* = 0.788
		MP 1500 mg/day	0.65 ± 0.09 F (1,28) = 0.905, *P* = 0.608	0.66 ± 0.12 F (1,28) = 0.195, *P* = 0.550

Sodium (mEq/L)	130–147 mEq/L	Placebo	140.47 ± 2.00	138.20 ± 2.60
		MP 50 mg/day	140.80 ± 1.66 F (1,28) = 0.400, *P* = 0.527	133.40 ± 18.73 F (1,28) = 0.256, *P* = 0.613
		MP 1500 mg/day	139.67 ± 2.13 F (1,28) = 0.303, *P* = 0.992	138.27 ± 2.46 F (1,28) = 0.319, *P* = 0.852

Potassium (mEq/L)	3.4–4.7 mEq/L	Placebo	4.67 ± 0.24	4.78 ± 0.57
		MP 50 mg/day	4.69 ± 0.38 F (1,28) = 0.421, *P* = 0.517	5.03 ± 0.68 F (1,28) = 1.216, *P* = 0.270
		MP 1500 mg/day	4.71 ± 0.62 F (1,28) = 0.822, *P* = 0.663	4.97 ± 0.55 F (1,28) = 1.444, *P* = 0.468

Chloride (mEq/L)	96–107 mEq/L	Placebo	94.70 ± 23.34	94.32 ± 23.24
		MP 50 mg/day	100.73 ± 1.22 F (1,28) = 0.550, *P* = 0.815	100.47 ± 1.51 F (1,28) = 0.542, *P* = 0.461
		MP 1500 mg/day	100.27 ± 2.79 F = (1,28) = 0.514 *P* = 0.773	101.33 ± 2.38 F = (1,28) = 1.872 *P* = 0.392

Cholesterol (mg/dL)	127–262 mg/dL	Placebo	214.00 ± 50.96	195.53 ± 48.86
		MP 50 mg/day	217.27 ± 48.77 F (1,28) = 0.032, *P* = 0.859	208.07 ± 42.55 F (1,28) = 0.362, *P* = 0.547
		MP 1500 mg/day	266.80 ± 32.03 F (1,28) = 0.215, *P* = 0.647	203.44 ± 24.42 F (1,28) = 0.650, *P* = 0.723

Albumin (g/dL)	3.8–5.4 g/dL	Placebo	3.90 ± 1.41	4.46 ± 040
		MP 50 mg/day	3.52 ± 1.59 F (1,28) = 0.789, *P* = 0.382	4.19 ± 0.02^∗^ F (1,28) = 6.404, *P* = 0.010
		MP 1500 mg/day	3.13 ± 1.63 F (1,28) = 0.050, *P* = 0.824	4.41 ± 26^∗^ F (1,28) = 8.348, *P* = 0.015

Globulin (g/dL)	2.6–3.4 g/dL	Placebo	2.69 ± 0.90	2.83 ± 0.35
		MP 50 mg/day	2.42 ± 1.05 F (1,28) = 0.520, *P* = 0.477	3.01 ± 0.44 F (1,28) = 2.718, *P* = 0.099
		MP 1500 mg/day	2.14 ± 1.08 F (1,28) = 0.058, *P* = 0.811	2.95 ± 0.28 F (1,28) = 3.079, *P* = 0.215

Bilirubin total (mg/dL)	0.3–1.5 mg/dL	Placebo	0.61 ± 0.39	0.51 ± 0.12
		MP 50 mg/day	0.67 ± 0.35 F (1,28) = 0.000, *P* = 0.983	0.49 ± 0.16 F (1,28) = 0.776, *P* = 0.387
		MP 1500 mg/day	0.66 ± 0.36 F (1,28) = 0.246, *P* = 0.881	0.51 ± 0.24 F (1,28) = 0.566, *P* = 0.754

Bilirubin direct (mg/dL)	0–0.5 mg/dL	Placebo	0.21 ± 0.09	0.19 ± 0.09
		MP 50 mg/day	0.21 ± 0.09 F (1,28) = 0.178, *P* = 0.673	0.16 ± 0.07 F (1,28) = 0.741, *P* = 0.387
		MP 1500 mg/day	0.20 ± 0.10 F (1,28) = 0.276, *P* = 0.871	0.15 ± 0.09 F (1,28) = 1.233, *P* = 0.540

Alanine aminotransferase or ALT (U/L)	4–36 U/L	Placebo	21.93 ± 10.85	26.33 ± 13.71
		MP 50 mg/day	16.67 ± 7.21 F (1,28) = 2.005, *P* = 0.157	18.53 ± 9.04 F (1,28) = 3.492, *P* = 0.062
		MP 1500 mg/day	17.00 ± 5.54 F (1,28) = 2.129, *P* = 0.345	18.87 ± 5.60 F (1,28) = 4.192, *P* = 0.123

Aspartate aminotransferase or AST (U/L)	12–32 U/L	Placebo	22.00 ± 5.01	24.93 ± 7.27
		MP 50 mg/day	19.93 ± 3.79 F (1,28) = 0.919, *P* = 0.338	22.20 ± 7.31 F (1,28) = 1.716, *P* = 0.190
		MP 1500 mg/day	24.40 ± 6.45 F (1,28) = 0.934, *P* = 0.623	23.93 ± 7.45 F (1,28) = 2.329, *P* = 0.312

Lactic acid dehydrogenase or LDH (U/L)	89–221 U/L	Placebo	214.07 ± 42.39	218.50 ± 49.20
		MP 50 mg/day	202.40 ± 37.44 F (1,28) = 0.990, *P* = 0.340	219.54 ± 36.49 F (1,28) = 0.097, *P* = 0.756
		MP 1500 mg/day	197.38 ± 24.93 F (1,28) = 0.879, *P* = 0.391	219.67 ± 47.83 F (1,28) = 0.586, *P* = 0.743

Creatine kinase–MB or CK-MB (U/L)	0–25 U/L	Placebo	19.30 ± 5.12	18.53 ± 5.05
		MP 50 mg/day	18.91 ± 6.34 F (1,28) = 1.781, *P* = 0.182	21.53 ± 5.90 F (1,28) = 1.883, *P* = 0.170
		MP 1500 mg/day	17.71 ± 7.51 F (1,28) = 1.879, *P* = 0.396	20.53 ± 9.20 F (1,28) = 1.657, *P* = 0.437

Triglyceride (mg/dL)	10–200 mg/dL	Placebo	141.53 ± 57.48	148.60 ± 54.76
		MP 50 mg/day	137.53 ± 62.04 F (1,28) = 2.755, *P* = 0.097	97.20 ± 45.88^∗∗^ F (1,28) = 8.434, *P* = 0.004
		MP 1500 mg/day	117.93 ± 48 F (1,28) = 3.092, *P* = 0.213	119.07 ± 50.23^∗^ F (1,28) = 8.792, *P* = 0.011

High density lipoprotein-cholesterol or HDL-C (mg/dL)	>35 mg/dL	Placebo	48.79 ± 17.40	52.07 ± 9.18
		MP 50 mg/day	41.95 ± 16.44 F (1,28) = 2.90, *P* = 0.089	60.27 ± 12.06 F (1,28) = 0.491, *P* = 0.045
		MP 1500 mg/day	36.93 ± 16.46 F (1,28) = 2.571, *P* = 0.276	64.47 ± 18.80^∗^ F (1,28) = 5.267, *P* = 0.029

Low density lipoprotein-cholesterol or LDL-cholesterol (mg/dL)	0–150 mg/dL	Placebo	121.49 ± 40.8	141.80 ± 24.29
		MP 50 mg/day	109.19 ± 47.47 F (1,28) = 0.007, *P* = 0.934	156.20 ± 47.27 F (1,28) = 0.001, *P* = 0.980
		MP 1500 mg/day	100.82 ± 51.02 F (1,28) = 0.107, *P* = 0.948	141.47 ± 24.23 F (1,28) = 0.001, *P* = 1.000

**Table 3:** Effect of various doses of the herbal congee containing the combined extract of *M. alba* leaves and *P. odoratum* (MP) on the hematological parameters (*N* = 45). Values are expressed as mean ± SD.

CBC	Normal value	Group	Baseline	8 weeks
			Mean ± SD	Mean ± SD
Red blood cell or RBC (x 10^6^/*μ*L)	4.0–5.20 (x 10^6^/*μ*L)	Placebo	3.92 ± 1.71	4.13 ± 1.33
		MP 50 mg/day	3.15 ± 1.97 F (1,28) = 1.284, *P* = 0.267	3.65 ± 1.62 F (1,28) = 2.048, *P* = 0.163
		MP 1500 mg/day	2.69 ± 1.03 F (1,28) = 2.138, *P* = 0.155	3.19 ± 1.77 F (1,28) = 1.845, *P* = 0.185

Hemoglobin or HGB (g/dL)	12.0–14.3 g/dL	Placebo	10.87 ± 4.91	10.99 ± 3.69
		MP 50 mg/day	8.41 ± 5.19 F (1,28) = 0.208, *P* = 0.648	10.12 ± 4.49 F (1,28) = 0.761, *P* = 0.383
		MP 1500 mg/day	7.35 ± 2.84 F (1,28) = 1.215, *P* = 0.545	8.34 ± 4.49 F (1,28) = 0.947, *P* = 0.623

Hematocrit or HCT (%)	36.0–47.7%	Placebo	32.34 ± 14.70	36.58 ± 12.48
		MP 50 mg/day	25.10 ± 15.77 F (1,28) = 1.399, *P* = 0.237	33.95 ± 15.42 F (1,28) = 0.291, *P* = 0.590
		MP 1500 mg/day	21.98 ± 8.34 F (1,28) = 0.862, *P* = 0.239	29.48 ± 16.79 F (1,28) = 0.323, *P* = 0.851

White blood cells or WBC (x 10^3^/*μ*L)	4.60–10.60 x 10^3^/*μ*L	Placebo	6.19 ± 3.25	5.41 ± 1.91
		MP 50 mg/day	4.65 ± 1.99 F (1,28) = 0.950, *P* = 0.330	5.66 ± 1.15 F (1,28) = 0.692, *P* = 0.329
		MP 1500 mg/day	4.02 ± 1.87	5.94 ± 1.13

Platelet or PLT (x 10^3^/*μ*L)	173–383 x 10^3^/*μ*L	Placebo	248.01 ± 105.23 F (1,28) = 1.049, *P* = 0.592	274.20 ± 85.65 F (1,28) = 4.229, *P* = 0.409
		MP 50 mg/day	183.86 ± 95.34 F (1,28) = 1.304, *P* = 0.263	245.65 ± 104.49 F (1,28) = 0.071, *P* = 0.792
		MP 1500 mg/day	158.11 ± 71.84 F (1,28) = 0.134, *P* = 0.717	206.37 ± 108.42^∗^ F (1,28) = 6.935, *P* = 0.031

Mean platelet volume or MPV (fl)	8.5–12.8 fl	Placebo	7.28 ± 3.07	8.10 ± 2.55
		MP 50 mg/day	5.65 ± 3.39 F (1,28) = 0.000, *P* = 0.983	7.07 ± 3.02 F (1,28) = 0.070, *P* = 0.935
		MP 1500 mg/day	4.85 ± 1.99 F (1,28) = 0.218, *P* = 0.897	5.96 ± 3.20 F (1,28) = 0.008, *P* = 0.927

Neutrophil or NE (%)	43.7–70.9%	Placebo	48.36 ± 21.50	42.54 ± 14.71
		MP 50 mg/day	37.54 ± 20.67 F F (1,28) = 0.001, *P* = 0.971	37.56 ± 16.97 F (1,28) = 0.292, *P* = 0.593
		MP 1500 mg/day	32.02 ± 13.38 F (1,28) = 0.002, *P* = 0.966	33.72 ± 17.88 F (1,28) = 0.155, *P* = 0.337

Lymphocyte or LY (%)	20.1–44.5%	Placebo	29.21 ± 13.61	37.29 ± 12.32
		MP 50 mg/day	23.96 ± 12.03 F (1,28) = 0.035, *P* = 0.852	33.91 ± 15.01 F (1,28) = 0.108, *P* = 0.744
		MP 1500 mg/day	19.70 ± 8.25 F (1,28) = 0.032, *P* = 0.859	27.70 ± 14.12 F (1,28) = 0.267, *P* = 0.609

Monocyte or MO (%)	3.4–9.8%	Placebo	5.78 ± 1.95	6.10 ± 1.92
		MP 50 mg/day	4.88 ± 2.71 F (1,28) = 1.00, *P* = 0.326	5.26 ± 2.06 F (1,28) = 0.466, *P* = 0.500
		MP 1500 mg/day	3.83 ± 1.80 F (1,28) = 0.037, *P* = 0.848	4.44 ± 2.12 F (1,28) = 0.005, *P* = 0.942

Eosinophil or EO (%)	0.7–9.2%	Placebo	3.49 ± 1.97	5.16 ± 3.23
		MP 50 mg/day	3.24 ± 0.89 F (1,28) = 1.765 *P* = 0.184	4.72 ± 3.02 F (1,28) = 1.255, *P* = 0.263
		MP 1500 mg/day	3.40 ± 1.21 F (1,28) = 2.131, *P* = 0.344	3.82 ± 8.09 F (1,28) = 1.788, *P* = 0.409

Basophil or BA (%)	0.0–2.6%	Placebo	0.44 ± 0.21 F (1,28) = 0.757, *P* = 0.384	0.78 ± 0.47 F (1,28) = 0.373, *P* = 0.545
		MP 50 mg/day	0.38 ± 0.17 F (1,28) = 0.519, *P* = 0.477	0.68 ± 0.42 F (1,28) = 373, *P* = 0.541
		MP 1500 mg/day	0.29 ± 0.12 F (1,28) = 0.911, *P* = 0.348	0.62 ± 0.29 F (1,28) = 0.794, *P* = 0.672

Mean corpuscular volume or MCV (fl)	80.0–97.8 fl	Placebo	71.33 ± 31.11	78.58 ± 25.49
		MP 50 mg/day	54.06 ± 31.73 F (1,28) = 0.363, *P* = 0.548	70.17 ± 31.05 F (1,28) = 0.450, *P* = 0.529
		MP 1500 mg/day	47.06 ± 19.38 F (1,28) = 0.517, *P* = 0.772	56.01 ± 28.70 F (1,28) = 0.553, *P* = 0.463

Mean corpuscular hemoglobin MCH (pg)	25.2–32.0 pg	Placebo	23.98 ± 10.43	25.39 ± 8.20
		MP 50 mg/day	18.10 ± 10.34 F (1,28) = 2.559, *P* = 0.110	22.81 ± 10.10 F (1,28) = 0.100, *P* = 0.922
		MP 1500 mg/day	15.71 ± 6.60 F (1,28) = 0.273, *P* = 0.873	18.25 ± 9.39 F (1,28) = 0.219, *P* = 0.644

Mean corpuscular hemoglobin concentration or MCHC (g/dL)	31.3–33.4 g/dL	Placebo	29.97 ± 13.75	29.30 ± 10.07
		MP 50 mg/day	22.75 ± 13.62 F (1,28) = 3.604, *P* = 0.068	26.18 ± 12.08 F F (1,28) = 1.533, *P* = 0.226
		MP 1500 mg/day	20.02 ± 7.89 F (1,28) = 0.992, *P* = 0.328	22.02 ± 12.56 F (1,28) = 0.003, *P* = 0.959

Red blood cells distribution width or RDW (%)	11.9–14.8%	Placebo	12.57 ± 3.49	12.51 ± 3.96
		MP 50 mg/day	10.16 ± 5.27 F (1,28) = 0.011, *P* = 0.917	10.98 ± 4.60 F (1,28) = 3.822, *P* = 0.051
		MP 1500 mg/day	7.87 ± 4.22 F (1,28) = 2.650, *P* = 0.266	9.25 ± 4.89 F (1,28) = 0.170, *P* = 0.075

## Data Availability

The data used to support the findings of this study are available from the corresponding author upon request.
